# Enzyme-Linked Immunosorbent Assay-Format Tissue Culture Infectious Dose-50 Test for Titrating Dengue Virus

**DOI:** 10.1371/journal.pone.0022553

**Published:** 2011-07-25

**Authors:** Jie Li, Dong-mei Hu, Xi-xia Ding, Yue Chen, Yu-xian Pan, Li-wen Qiu, Xiao-yan Che

**Affiliations:** 1 Center for Clinical Laboratory, Zhujiang Hospital,Southern Medical University, Guangzhou, People's Republic of China; 2 Key Laboratory of Prevention and Control of Emerging Infectious Diseases of Guangdong Higher Education Institutes, Guangzhou, People's Republic of China; Universidade Federal do Rio de Janeiro, Brazil

## Abstract

A dengue nonstructural protein 1 (NS1) antigen capture enzyme-linked immunosorbent assay (ELISA)-based tissue culture infectious dose-50 (TCID_50_) test (TCID_50_-ELISA) was developed as an alternative to the standard plaque assay for titrating dengue virus. Virus titers obtained by TCID_50_-ELISA were comparable to those obtained by the plaque assay and by the traditional TCID_50_-cytopathic effect (CPE) test (TCID_50_-CPE), with a better reproducibility and a lower coefficient of variation. Quantitative comparison of TCID_50_-ELISA and TCID_50_-CPE resulted in a correlation coefficient of 0.976. Moreover, this new method showed a wider application to C6/36, Vero E6, BHK-21, and Vero cells compared with other titration methods. In summary, the novel TCID_50_-ELISA method described here provides a more reliable and more accurate alternative compared to the plaque assay and TCID_50_-CPE for titration of dengue virus.

## Introduction

Over the past 60 years, dengue fever and dengue haemorrhagic fever have become increasingly serious public health problems in the tropics and subtropics due to overpopulation, ever-increasing regional and international travel, as well as global warming [1]. Dengue virus (DENV), a member of the family *Flaviviridae*, genus *Flavivirus*, is an enveloped, single-stranded RNA virus comprising four antigenically distinct serotypes: DENV1, DENV2, DENV3, and DENV4. Infection with any one of the four serotypes can result in a broad spectrum of consequences, including asymptomatic infection, mild febrile illness, classic dengue fever, and the lethal dengue haemorrhagic fever (DHF)/dengue shock syndrome (DSS) [2]. Research on DENV is often hindered by inefficient and inaccurate or costly viral titration methods [3]. Hence, a simple and efficient assay for accurate titration of DENV in infected cultures would greatly facilitate dengue research, vaccine development, and laboratory detection. To date, a variety of methods for titrating DENV have been developed, including classical assays, the plaque assay and the tissue culture infectious dose-50 assay (TCID_50_), and immunofluorescence-based assays such as fluorescence-activated cell sorting (FACS) assay and fluorescent focus assay [4], [5], [6], [7]. As a standard method for titrating DENV, however, the plaque or TCID_50_ assays have their disadvantages, as they are limited to some strains and passages of the virus, and a few cell lines [5]. Most primary clinical isolates do not form clear plaques or have a visible cytopathic effect (CPE) on cell monolayers. Furthermore, both of these assays require manual microscope examination daily, which is time consuming and labour intensive. FACS and fluorescent focus assays can provide more rapid and accurate quantitation of DENV than the traditional plaque assay [4], [6]. However, each of these techniques requires experienced technicians and sophisticated laboratories, hindering its application in most laboratories lacking sophisticated equipment. Therefore, convenient methods for titrating the virus need to be developed. Nonstructural protein 1 (NS1), a multifunctional glycoprotein in dengue virus, is highly conserved for all serotypes of DENV and is strongly immunogenic [8]. Some of the NS1 protein is expressed as a soluble secreted form, which has been implicated to contribute to dengue viral propagation and the amount secreted is closely related to dengue viral titer [9]. In our previous study, we established a dengue NS1 antigen capture enzyme-linked immunosorbent assay (ELISA) [10]. In the present study, a novel TCID_50_ assay was developed, which employs this dengue NS1 antigen capture ELISA for the accurate and objective titration of DENV.

## Materials and Methods

### Cells and viruses

*Aedes albopictus* cells (C6/36, ATCC:CRL-1660) were maintained in minimum essential medium (MEM; Gibco) supplemented with 10% fetal bovine serum (FBS; HyClone Laboratories, Logan, Utah) and 0.1% gentamicin (50 mg/mL, Sigma Chemical Co., St. Louis, Mo.) at 28°C or 37°C in 5% CO_2_. African green monkey kidney cells (Vero E6, ATCC:CRL-1586), Hamster kidney cells (BHK-21, ATCC:CCL-10) and African green monkey kidney cells (Vero, ATCC:CCL-81) were maintained in MEM supplemented with 10% FBS (Gibco), 0.1% gentamicin (50 mg/mL) at 33°C in 5% CO_2_.

DENV serotypes used in TCID_50_-CPE, TCID_50_-ELISA and plaque assay were as follows: DENV1, Hawaii; DENV2, New Guinea-C; DENV3, Guanxi-80-2; DENV4, H241. The four serotypes of DENV were obtained from the Center for Disease Control and Prevention of Guangzhou, China. The virus working stocks were prepared by growing in C6/36 cells at 33°C in 5% CO_2_ for 3 to 5 days. After the cytopathic effect (CPE) was observed, cell culture supernatant was collected, clarified by centrifugation, supplemented with 20% heat-inactivated FBS, and stored in aliquots at −80°C.

### Plaque assay in Vero E6 cells

The plaque assay in Vero E6 cells was performed using a protocol modified from Roehrig [11]. Briefly, Vero E6 cells were seeded in six-well plates at a density of 4×10^5^ cells/well and incubated overnight at 33°C in 5% CO_2_ until cells were approximately 80% to 90% confluent. Medium was removed from cells, and replaced with 500 µl of medium (MEM with 2% FBS) containing 10-fold serial dilutions of virus. Viral adsorption was allowed to proceed for 90 min at 33°C with rocking of the plates every 15 min. The supernatant was aspirated off gently and the monolayers were overlaid with 3 mL per well of MEM containing 2% FBS, 1.2% carboxymethyl cellulose (Sigma) and 0.1% gentamicin (50 mg/mL). Plates were incubated at 33°C in 5% CO_2_ for 5 to 7 days or until the CPE was observed. Next, the nutrient agar overlay was removed and the cells were fixed with 4% formalin for 1 hour. Staining solution (1 mL per well) containing 0.8% crystal violet (Sigma) was added then removed after 15 min. Plates were rinsed and dried at room temperature, and plaques were counted immediately. The assay was carried out in triplicate and the plaque counts were averaged. The viral titers were expressed as plaque-forming units (PFU)/mL, calculated as [(number of plaques per well)×(dilution)]/(inoculum volume).

### TCID_50_-CPE

TCID_50_-CPE was performed using a protocol modified from Schoepp and Beaty [12]. Briefly, cells were seeded in a 96-well plate at a density of 2.5×10^5^ cells/well (C6/36 cells) or 1×10^4^ cells/well (Vero E6, BHK-21 or Vero cells) in 200 µL and incubated overnight at 28°C (37°C for mammalian cells) in 5% CO_2_. Ten-fold serial dilutions of the virus were prepared in MEM (10^−1^–10^−10^) and 100 µL of each dilution was added to 10 seeded wells. Viral adsorption was allowed to proceed for 90 min at 33°C. The supernatant was aspirated off gently and the monolayers were overlaid with 200 µL per well of MEM with 0.1% gentamicin. Ten negative control wells without virus were included in each plate, and the CPE was observed daily using an inverted microscope. The 50% tissue culture infectious dose (TCID_50_) of each virus was calculated as described by Reed and Muench (1938) when no further CPE was observed [13].

### TCID_50_-ELISA

C6/36, Vero E6, Vero, or BHK-21 cells were seeded in 96-well plates at an appropriate density. The TCID_50_-CPE assay was performed to titrate DENV1 to 4 as described above. After an appropriate incubation period, the NS1 protein in 100 µL of culture supernatant from each well was determined using a dengue NS1 antigen capture ELISA method established previously in our laboratory [10]. The optical densities (ODs) were read at 450 nm using SureBlue Reserve™ TMB Microwell Peroxidase Substrate (KPL) containing 3,3′,5,5′ tetramethylbenzidine in a mildly acidic buffer for color development. The cutoff value was established as the 2.1-fold of the mean OD value of control cells without virus. The CPE of each microtiter plate was read by two investigators independently just before the ELISA test. The final titer of TCID_50_-CPE was defined as the mean value of the titers obtained by both investigators, and the results of TCID_50_-CPE read by each investigator were compared with the corresponding results of TCID_50_-ELISA.

### Determination of incubation time for TCID_50_-ELISA

To determine the optimal incubation time for titrating DENV 1 to 4 using TCID_50_-ELISA, we conducted the TCID_50_-CPE test in C6/36 and Vero E6 cells as described above. The plates were incubated for 2, 4, 6, 8, 10, 15, or 20 days. After incubation, TCID_50_-ELISA was performed to determine the NS1 protein in 100 µL of culture supernatant from each well. Each test was run in triplicate.

### Data analysis

All statistical analyses were performed using statistical package version SPSS 13.0. Kruskal-Wallis test, Friedman's test and Wilcoxon's signed rank test were used to evaluate the statistical significance of the results. A *P* value<0.05 was considered statistically significant for all parameters.

## Results

### Determination of incubation time for TCID_50_-ELISA assay

**Figure 1:**
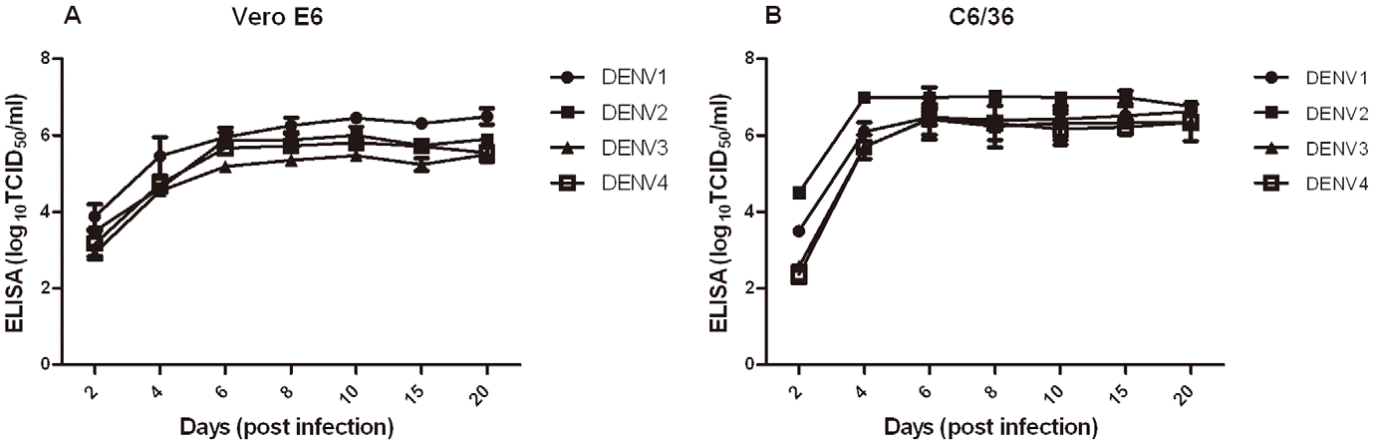
Determination of the incubation time for titration of DENV 1 to 4 by TCID_50_-ELISA. Supernatants for TCID_50_ tests from Vero E6 cells (A) and C6/36 cells (B), which had been exposed to DENV1 to 4 for 2, 4, 6, 8, 10, 15, 20 days, were collected and analyzed for NS1 production by ELISA. Data are averaged from 3 separate experiments. Results are shown as means ± standard deviations.

TCID_50_-ELISA assays were conducted with C6/36 and Vero E6 cell lines for optimization of the incubation time (**Table 1**). After incubation for 2, 4, 6, 8, 10, 15, or 20 days, significant differences between the TCID_50_-ELISA titers of DENV 1 to 4 were observed (Kruskal-Wallis test, *P* = 0.000). However, as shown in **Figure 1**, the TCID_50_-ELISA titers increased significantly up to day 6 and then remained stable up to day 20. Additionally, the mean rank value of TCID_50_-ELISA titers obtained by day 6 was very similar to the results obtained over the next 4 time points (Kruskal-Wallis test). Hence, the 6 day incubation appeared to be sufficient for the infected cells to produce enough NS1 protein to be detected by ELISA, and this assay should be applicable to cultures at anytime between 6 and 20 days.

**Table 1:** Titration of DENV 1 to 4 by TCID_50_-ELISA and TCID_50_-CPE in Vero E6 and C6/36 cells, at seven different incubation times.

Virus	Incubation times (days)	Vero E6		C6/36	
		ELISA titer^a^	CPE titer	ELISA titer	CPE titer
DENV1	2	3.9±0.5	n/a^b^	3.5±0.0	n/a
	4	5.5±0.9	4.5±1.0	6.1±0.2	2.5±0.0
	6	6.0±0.4	5.9±0.5	6.5±0.2	n/a
	8	6.3±0.4	5.9±0.4	6.4±0.0	n/a
	10	6.5±0.2	6.1±0.5	6.4±0.2	n/a
	15	6.3±0.2	6.1±0.3	6.5±0.2	n/a
	20	6.5±0.4	6.3±0.5	6.6±0.1	n/a
DENV2	2	3.5±0.0	n/a	4.5±0.0	n/a
	4	4.6±0.0	4.6±0.1	7.0±0.1	5.2±0.6
	6	5.9±0.3	5.7±0.4	7.0±0.3	n/a
	8	5.9±0.3	5.6±0.4	7.0±0.2	n/a
	10	6.0±0.4	5.5±0.4	7.0±0.1	n/a
	15	5.7±0.2	5.5±0.5	7.0±0.2	n/a
	20	5.9±0.2	5.6±0.3	6.8±0.1	n/a
DENV3	2	2.9±0.3	n/a	2.6±0.1	n/a
	4	4.6±0.1	3.5±1.0	5.7±0.1	n/a
	6	5.2±0.2	4.5±0.1	6.4±0.4	4.7±1.0
	8	5.4±0.1	4.6±0.2	6.2±0.5	5.4±0.1
	10	5.5±0.1	4.7±0.3	6.3±0.4	6.0±0.2
	15	5.2±0.3	4.8±0.3	6.3±0.1	6.1±0.2
	20	5.5±0.3	5.1±0.3	6.3±0.1	6.4±0.1
DENV4	2	3.2±0.6	n/a	2.4±0.2	n/a
	4	4.7±0.4	4.8±0.6	5.7±0.3	n/a
	6	5.7±0.1	5.7±0.1	6.4±0.6	5.3±0.8
	8	5.7±0.2	5.6±0.1	6.3±0.4	5.5±1.0
	10	5.8±0.2	5.7±0.2	6.2±0.4	5.4±0.9
	15	5.7±0.1	5.6±0.1	6.2±0.2	6.0±0.4
	20	5.5±0.1	5.5±0.1	6.3±0.5	6.2±0.4

a Geometric mean titer (titers are expressed as log_10_TCID_50_ per milliliter). Each number represents the mean of three tests ± standard deviation.

b n/a = not applicable. CPE did not develop or could not be distinguished from control cells.

### Qualitative and quantitative comparisons of TCID_50_-ELISA, TCID_50_-CPE and plaque assay

#### Accuracy

**Figure 2:**
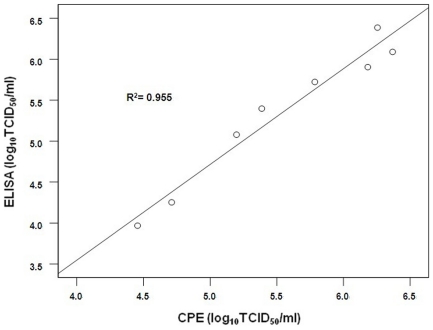
Correlation between virus titers obtained by TCID_50_-CPE and TCID_50_-ELISA of DENV1 to 4. R^2^ = 0.955. Data are from 12 separate titrations with each virus titered three times.

The accuracy of the TCID_50_-ELISA assay was evaluated by comparing the titers from TCID_50_-ELISA with those from TCID_50_-CPE, which is one of the classic methods for DENV titration. Serial 10-fold dilutions of DENV were inoculated into four susceptible cell lines (C6/36, Vero E6, Vero, and BHK-21), TCID_50_ assays were performed, and CPE was observed and recorded daily. Although 6 days of incubation is enough for the TCID_50_-ELISA as described above, 10 days was chosen for these TCID_50_ assays because CPE in Vero or BHK-21 cells was not observed at earlier time points (data not shown). After 10 days of incubation, 100 µL of supernatant from each well was aspirated and assayed for the NS1 antigen using our ELISA method. No significant differences were observed between the titers of DENV obtained by TCID_50_-ELISA and by TCID_50_-CPE in C6/36 (Friedman test, *P* = 0.068), Vero E6 (*P* = 0.465), Vero (*P* = 0.068), or BHK-21 cells (*P* = 0.273). Moreover, the titers of DENV obtained by TCID_50_-ELISA or TCID_50_-CPE in different cells were significantly different (Friedman test; *P* = 0.026 and *P* = 0.011, respectively). A good agreement was observed between the titers from TCID_50_-CPE and TCID_50_-ELISA, with a correlation coefficient of 0.976 (R^2^ = 0.955, *P* = 0.000, **Figure 2**). It was noted that the TCID_50_-CPE titers sometimes could not be obtained accurately because of nonspecific changes in cell cultures due to non-optimal growth medium or cell aging. These results demonstrate high accuracy and reliability of the TCID_50_-ELISA assay, and also showed that it has a wider application than TCID_50_-CPE because the results appeared to be unaffected by the state of cell line used.

#### Reproducibility

Reproducibility of the TCID_50_-ELISA assay was evaluated in several ways. First, the titers of DENV obtained by TCID_50_-ELISA, TCID_50_-CPE and plaque assay were analyzed, and their coefficients of variation (CVs) were compared. The titers of the TCID_50_-ELISA assay exhibited the smallest CV of 0.068, followed by those of the TCID_50_-CPE assay (CV = 0.099). The plaque assay had the highest CV (CV = 0.272). When the same dilutions of DENV were inoculated in C6/36, Vero E6, Vero, or BHK-21 cells in a parallel plaque assay, suitable plaques developed in Vero E6 cells but not in other three cell lines. Thus, the plaque assay is applicable only to particular cell lines. Comparison of the CVs from the four cell lines showed no significant differences between the results obtained from TCID_50_-ELISA and TCID_50_-CPE (Wilcoxon signed ranks test). In addition, there were no significant differences between the CVs from the four cell lines when tested by TCID_50_-ELISA or by TCID_50_-CPE (Friedman test; *P* = 0.825 and *P* = 0.552, respectively). All these results showed that the TCID_50_-ELISA assay has a higher degree of reproducibility and is applicable to a wider range of cell lines than the plaque assay and the TCID_50_-CPE assay.

## Discussion

Dengue viruses are spherical, lipid-enveloped viruses that contain a positive strand RNA genome of approximately 10,200 nucleotides coding for three structural proteins (capsid, membrane, and envelope) and seven nonstructural proteins (NS1, NS2a, NS2b, NS3, NS4a, NS4b, NS5) [14]. NS1, as a key marker of ongoing dengue viral replication, is secreted into the extracellular fluid and is also expressed on the surface of infected cells [15]. A novel TCID_50_ assay is described for determination of dengue virus titers, which relies on detecting the DENV NS1 protein by ELISA. In this TCID_50_-ELISA assay, the observation of the CPE was replaced by determination of NS1 protein, which can eliminate subjective variations between operators and laboratories. With this method, we were able to run large numbers of titration tests and to automate reading and recording of the data.

This novel TCID_50_-ELISA method has several advantages over the plaque assay and the TCID_50_-CPE assay. A major advantage of the TCID_50_-ELISA assay is the ability to quantitate viruses using cell lines in which a CPE is not detectable or plaques are not visible. This widens the range of cell lines in which viral titer can be detected. For instance, viral titers were obtained for DENV 1 to 4 in BHK-21 and Vero cells, in which a CPE was not observed. Moreover, the results of the TCID_50_-ELISA assay described here can be recorded automatically from the plate in which the test is carried out. This simplifies the test procedure and eliminates the fatigue and subjectivity associated with reading large numbers of tests in the CPE analysis. Furthermore, the TCID_50_-ELISA assay showed better reliability because the ELISA results are not subject to nonspecific changes in cells, which can make CPE reading difficult. Finally, the TCID_50_-ELISA assay has been used successfully for clinically DENV isolates, which might not be assessable by other titration methods (data not shown). In conclusion, an efficient assay for titration of DENV is reported. The method was validated by comparison with data obtained by other techniques. This TCID_50_-ELISA assay overcomes the flaws inherent in the plaque assay and the TCID_50_-CPE assay. Based on its reliability and ease of execution, the TCID_50_-ELISA test represents a promising assay for titration of DENV, and will facilitate dengue research.
